# Visible Goiter among Pregnant Women Attending Antenatal Clinic in Public Health Facilities of Debre Markos Town, East Gojjam, North West Ethiopia

**DOI:** 10.1155/2019/2484523

**Published:** 2019-12-23

**Authors:** Yihun Endalamaw, Haji Kedir, Tadesse Alemayehu

**Affiliations:** ^1^Medicins Sans Frontiers, Addis Ababa, Ethiopia; ^2^Haramaya University, College of Health & Medical Sciences, P.O. Box 235, Harar, Ethiopia

## Abstract

**Background:**

Goiter is an abnormal enlargement of the thyroid gland due to inadequate intake of iodine and goitrogenic food. It is the most important public health problem in developing countries like Ethiopia and specifically in East Gojjam. Though there are studies on goiter in Ethiopia, the magnitude is not well known and documented in Debre Markos town on pregnant women. Therefore, this study was carried out to assess the magnitude of visible goiter and associated factors among pregnant women visiting antenatal clinic in three public health facilities of Debre Markos town, North West Ethiopia.

**Methods:**

Facility-based cross-sectional study was conducted on 401 pregnant women visiting antenatal clinics at three public health facilities using the systematic sampling technique. Data were collected using pretested structured questionnaire by an interview method. All pregnant women were examined for the presence of goiter using World Health Organization (WHO) criteria. Both bivariate and multivariable binary logistic regression analyses were used to see the association between dependent and each independent variable.

**Result:**

The prevalence of visible goiter was found to be 10.5% (95% CI: 7.5–13.5). Visible goiter was more common in the age category between 15 and 19 years. Low household income (AOR = 4.5, 95% CI: 1.1–18.7), cabbage intake (AOR = 5.2, 95% CI: 1.2–22.3), and poor knowledge about the benefits of iodized salt (AOR = 2.4, 95% CI: 1.1, 5.2) were factors associated with visible goiter.

**Conclusion and Recommendation:**

Visible goiter is a major public health problem in this study area. Low socioeconomic status, low knowledge of pregnant women about the merits of iodized salt, and frequent intake of goitrogenic foods such as cabbage increase the risk of developing visible goiter. Therefore, due emphasis on goiter prevention and control strategies, increasing knowledge of women on the benefit of iodized salt, including low-income households in safety net programs, and nutritional education on iodine-rich diets (such as tuna, dairy products, and egg) should be emphasized to alleviate the problem.

## 1. Background

Goiter is one of the most pathological manifestations of thyroid gland disorder that indicates long-term depletion of iodine storage in the human body, particularly in pregnant women and children. It is a major public health threat mainly caused by inadequate intake of iodine-containing diets and high consumption of goitrogenic foods [1]. The problem is worse especially in populations living in the iodine-deficient environment and is more common in females and prevalence mainly depends on age, sex, iodine intake, diet (goitrogens), and therapeutic and environmental radiation exposure [2–4].

Globally, about 2,225 million people (38%) are at the risk of iodine deficiency disorder (IDD), while 740 million people (13%) are affected by goiter, highest prevalence in Eastern Mediterranean (32%) and Africa (20%) [5, 6], and contribute 2.72% of all sequelae of disease [7]. A total goiter rate (TGR) of 5% or more is recommended as the cutoff point to indicate a public health problem [1, 2].

Based on the nationwide nutrition baseline survey report for the national nutrition program of Ethiopia in 2010, around 28 million people are suffering from goiter, and more than 35 million people are at the risk of iodine deficiency [8]. About 6 million women in the reproductive age category were affected by goiter in Ethiopia [9]. It is one of the nations with the lowest consumption of iodized salt and the highest total goiter prevalence (36%) in Africa [10].

Goiter is still prevalent in different parts of the country especially in children and pregnant women of high land areas. The World Health Organization (WHO) recommends that studies should be conducted among pregnant women as these groups of the population are vulnerable to marginal iodine deficiency and are easily accessible during antenatal clinics, and the findings of the study can be inferred to the general community of the same characteristics [1]. Most goiter surveys have been done in school-age children or the general adult population and have only rarely included pregnant women. Thus, the aim of this study was to assess the magnitude of visible goiter and associated factors among pregnant women attending antenatal clinics in public health facilities of Debre Markos town, East Gojjam zone, North West Ethiopia.

## 2. Methods and Materials

### 2.1. Study Area and Design

An institutional-based cross-sectional study was conducted in March 2015 among pregnant women attending antenatal clinics at public health facilities of Debre Markos town.

### 2.2. Sample Size Determination

The sample size was calculated by both single and double population proportion formula. Then, the larger sample size was taken which was determined based on single population proportion using prevalence of 50% visible goiter rate, 5% marginal error, 95% confidence level, and 5% nonresponse rate. Accordingly, the result showed 404 pregnant women to be included in the study. Prevalence of 50% was used because we could not get literature from other areas with similar sociodemographic/economic characteristics of the current study area.

The sample was calculated by the following formula:

(1) $$\begin{array}{c} n=Z\left(\frac{\alpha }{2}\right)2\;*\;\frac{p\left(1-p\right)}{d^{2}}, \end{array}$$

where *n* = the desired sample size, *p* = assumed visible goiter rate in pregnant woman, *Z*(*α*/2) = critical value at 95% confidence level of certainty (1.96), and *d* = margin of error between the sample and population which is 5%.

### 2.3. Sampling Technique

Purposely, all the three public health facilities (inside the town) were included in the study, and the sample size was proportionally allocated to the three health facilities based on the average number of clients visited antenatal care at each facility over one month period prior to the study.

Pregnant women from each health facility were selected using a systematic sampling technique. Systematic sampling method was chosen because it was easy to get sampling frame. Accordingly, first, the total population (estimated pregnant women in the catchment areas for these health facilities) was divided by the required sample size to get the random start. Then, interview and physical examination was performed after selecting the first pregnant woman randomly from the first three who came for the antenatal visit.

### 2.4. Data Collection Methods and Instruments Used

Data were collected using a structured questionnaire translated to Amharic (local) language. The questionnaire has three parts. Part one was used to record the basic demographic characteristics of the respondents. Part two was used to measure factors contributing to the development of goiter. And lastly, part three contains checklists to evaluate goiter level.

Pregnant women were asked about their consumption patterns regarding goitrogenic foods (food items that foster the development of goiter) and those that prevent goiter. Their consumption frequency was requested how much they are consuming these food items on daily basis. In addition to this, iodized salt consumption rate was also assessed. They were asked about the type of salt they are using, ionized or not ionized. At the same time, the level of knowledge of women about ionized salt was also assessed. Authors did not do any laboratory tests, but women can easily identify by the package (usually unionized salt is not packed) whether they are using ionized salt or not.

Physical examination (inspection and palpation) of the thyroid gland was done to assess the goiter rate using the WHO/UNICEF/ICCIDD classification scheme [1]. Accordingly, the measurement was made into three grades; grade 0: none or no goiter; grade 1: palpable but not visible; grade 2: a swelling in the neck that is clearly visible when the neck is in a normal position and is consistent with an enlarged thyroid when the neck is palpated. During analysis, however, for the sake of using logistic regression and the purpose of this study was to assess visible goiter, and the three levels of grading were recorded in to two, i.e., grade 0 and 1 were recoded as no visible goiter (coded 0), and grade 2 was recoded as having visible goiter (coded 1).

### 2.5. Data Quality Control

The questionnaire was translated to the local language of the respondents. Training was given for data collectors, supervisors, and data clerks. The questionnaire was pretested on 10% of the sample size which was not included in the study before the actual study was conducted. Double data entry was done by two different data clerks to check consistency and to minimize errors.

### 2.6. Data Analysis

The data were entered into EpiData version 3.1 and analyzed using SPSS version 16. Descriptive summaries such as frequency and percentage were shown using tables and figures according to important characteristics of study participants.

Knowledge score of pregnant women on the benefit of iodized salt was calculated by asking six knowledge-related questions for every woman. Women who got the correct answer were scored 1 for each of the six knowledge-related questions, while those who did not answer correctly scored 0.

Bivariate analysis using binary logistic regression was made to see the crude association between the dependent and independent variables and the strength of association using the odds ratio along with 95% CI and *P* value. Multicollinearity between the independent variables was checked by a variance inflation factor (VIF), and model fitness test was done using Hosmer and Lemeshow test. Variables with the *P* value less than or equal to 0.25 were entered into multivariable analysis using multivariable logistic regression to see the effects of independent variables on visible goiter by controlling confounders. *P* value <0.05 level was used as a cutoff point to declare statistical significance.

### 2.7. Ethical Clearance

Ethical clearance and approval for the study were obtained from Institutional Health Research Ethical Review Committee of College of Health and Medical Sciences of Haramaya University. An official letter of cooperation was submitted to East Gojjam zonal health department. The district health office and three selected public health facilities were also asked with an official letter to get permission, and written informed consent was obtained from each study participant.

## 3. Results

### 3.1. Socioeconomic and Sociodemographic Characteristics

A total of 401 pregnant women were included making the response rate of 99.2%. The mean (±SD) age was found to be 27.2 ± 5.2 years, and most study participants, 144 (35.9%), lies in the age category between 25-29 years.

Two hundred and eighty-eight (71.8%) of study participants resided in Debre Markos town. About 139 (34.6%) study participants and 101 (25.2%) of their husbands were illiterate.

Among those included in the study, more than one-third (38.1%) of the respondents were housewife followed by governmental employee 130 (32.4%), and about 131 (32.7%) of the pregnant women had a monthly income of 1000–2000 Birr (1 $USD is equivalent to 28 Birr) (Table 1).

### 3.2. Knowledge Score of Study Participants

The mean knowledge score for all women was 4.2 (SD = 1.1) with a minimum score of 2 and a maximum score of 6. Overall, about 30.7% of pregnant women had poor knowledge about the benefits of iodized salt (Table 1).

Pregnant women dietary intake assessment showed that 322 (80.3%) of them consumed cabbage at least once per week. There is a statistical significance association between consumption of cabbage and development of goiter (*X*^2^ = 7.2, *P* < 0.05). With regard to iodized salt consumption, 240 (59.9%) reported they have consistently consumed it, and there was also a statistical significance relationship between consumption of iodized salt and development of goiter (*X*^2^ = 16.2, *P* < 0.05).

### 3.3. Prevalence of Goiter

**Figure 1:**
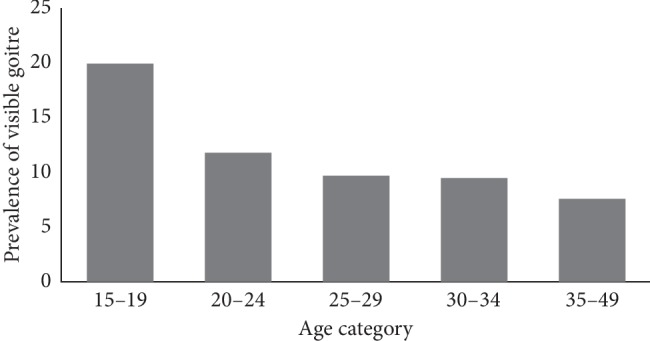
Visible goiter on pregnant women by age category in Debre Markos town, Northwest Ethiopia (*N* = 401).

This study finding showed that the total goiter rate (TGR) was found to be 39.4% (95% CI = 34.6–44.2), including 28.9% of palpable (Grade 1) and 10.5% of visible goiter (Grade 2). Analysis of visible goiter by age group showed a decrease in prevalence rates. It was highest (20%) for pregnant women within the age group of 15–19 years and lowest (8.3%) for those aged 35–49 years (Figure 1).

### 3.4. Factors Associated with Visible Goiter

All variables with *P* < 0.25 with the outcome variable in the bivariate analysis were included in the multivariable regression model. Multivariable binary logistic regression analysis revealed that household income, cabbage intake, and knowledge of pregnant women on the benefit of iodized salt were independently associated with visible goiter.

Women who were from low household income were at a higher risk of having visible goiter compared with their counterparts (AOR = 4.5; 95% CI = 1.1, 18.7). Pregnant women who consumed cabbage on a regular basis were significantly associated with the development of visible goiter compared with those who did not consume cabbage (AOR = 5.2; 95% CI = 1.2, 22.3). Visible goiter was more prevalent among pregnant women who had poor knowledge on the benefit of iodized salt compared with those who had good knowledge (AOR = 2.4; 95% CI 1.1, 5.2) (Table 2).

## 4. Discussion

This study assessed the magnitude of visible goiter and factors associated with it among pregnant women attending ANC at public health facilities. Accordingly, one out of ten women had a visible goiter. Household income, consumption of goitrogenic food such as cabbage, and knowledge of women on the benefits of iodized salt had an independent significant association with visible goiter.

In this study, the magnitude of visible goiter was 10.5%. This is consistent with a study conducted in the Amhara region of Ethiopia (8.6%) and the national prevalence of 11.5% [9]. However, a study conducted at Jimma University of Western Ethiopia among pregnant women showed that 31.5% of study participants had developed visible goiter which is higher than the current finding [11].

A study in Pakistan has also showed that visible goiter was about 21.4% among women which is twice higher than the finding of the current study [12]. This may be because the Pakistani study includes all women who came to health facility for goiter-related diagnosis which resulted in a higher prevalence.

The prevalence of visible goiter was highest at the younger age group of 15–19 years (20%) due to an increased metabolic demand of iodine during pregnancy and lowest (8.3%) above the age of 35 years. This finding is the same as studies conducted in other parts of Ethiopia: Burie and Womberma District of West Gojjam [8], Neksege subdistrict in Tigray [12], and in Pakistan [13] and China [14].

Pregnant women's income has shown a significant association with the development of visible goiter. Pregnant women whose monthly income less than 1000 Ethiopian Birr had a higher odds ratio of goiter prevalence than pregnant women whose monthly income is higher (AOR = 4.5 and 95% CI: 1.1–18.7). This finding is consistent with a study conducted in Jimma University of Western Ethiopia on pregnant women visiting antenatal clinics [11] and a study in the Kashimiri Region on reproductive age population (15–49 years) [15]. The higher figure in low-income status women might be due to poor nutritional intake including less meat, egg, milk, and fish products and use of locally available noniodized salt which is a common practice because it is not expensive as iodized salt. This, in turn, suggests that living standard has a direct relationship with iodine-rich diets and thereby the prevalence of visible goiter [11].

A relationship was found between cabbage intake and prevalence of visible goiter among the study population. Pregnant women who served/used cabbage on regular bases at least once a week were exposed to visible goiter more than those who had never taken cabbage (AOR = 5.2, 95% CI = 1.2–22.3, *P*=0.02). This is because cabbage is the cheapest, easily available, and locally grown vegetable in the study area. Cabbage contains thiocyanate and isothiocyanate that inhibit iodine uptake by the thyroid follicular cells and also blocks the thyroid peroxidase enzyme [16]. The present finding is supported by reports of the previous studies [11, 17, 18].

This study elucidated that there is a link between the knowledge of study participants on the merit of iodized salt consumption and enlargement of the thyroid gland. The odds of developing goiter were 2.4 times (AOR = 2.4; 95% CI = 1.1, 5.2) higher among those who had poor knowledge about the use of iodized salt than those who had good knowledge. In this study, about 69% of pregnant women had knowledge of the benefit of iodized salt. A study in Ghana also showed that 72% of the respondent had knowledge [19]. But, in a study conducted at Burie and Womberma District of Amhara region, Ethiopia, the knowledge of respondent on the benefit of iodized salt was very low [8]. This might be due to the fact that this study was done in the health facility, and respondents could get information while visiting antenatal clinics than the other study where it was done at a community level.

On the contrary, goitrogenic foods such as consumption of millet, sweet potato, and maize which were determinants of goiter in other studies [6, 12, 18] were not found to be significantly affecting the prevalence of visible goiter among pregnant women in the present study. Because these food items especially millet and sweet potato are not commonly found in the study area and are not used as staple food, and moreover their goitrogenic content lowers through cooking by stimulating the production of myrosinase, an enzyme that helps to deactivate goitrogenic glucosinolates [20, 21].

As strength, the findings of this study may raise the issue of policy concern for appropriate intervention programs and might serve as a monitoring and evaluation tool for salt iodization policy and could have a role in the assessment of long term impacts of control programs for goiter. Health care providers can use the findings as a guide to initiate appropriate interventions during prenatal, antenatal service provisions and to counsel clients to increase their knowledge on the use of iodized salt and intake of a variety of iodine-rich diets.

Besides its strength, the study might have some limitations. Dietary assessment of goitrogenic and iodine-rich foods intake based on dietary history might lack accuracy. Prevalence of visible goiter by inspection and palpation might face minimal interobserver variability and also lacks sensitivity to acute changes of iodine intake as it indicates long-term impact, and the findings might not indicate recent iodine intake. On the contrary, since the study is cross sectional and conducted in the dry season (seasonal variation), low availability of vegetables during a dry time may have an influence on the consumption of a variety of vegetables, and this could have affected the study. Furthermore, environmental (iodine content of soil or water), physiological (pregnancy), and genetic causes (genetically related diseases) may mask the result of the study as the current study did not explore them, hence needing further research.

## 5. Conclusions

The magnitude of visible goiter among pregnant women constitutes a major public health problem in the study area. One out of every ten pregnant women is at a risk of having visible goiter, and it is more common among pregnant women who are at young age groups. Low income, low knowledge of pregnant women about the merits of iodized salt, and frequent intake of goitrogenic foods such as cabbage are factors associated with visible goiter.

## Figures and Tables

**Table 1:** Sociodemographic characteristics and knowledge of pregnant women at public health facilities of Debre Markos town, Northwest Ethiopia (*N* = 401).

Variable	Category	Frequency	Percent (%)
Age	15–19	15	3.7
	20–24	119	29.7
	25–29	144	35.9
	30–34	63	15.7
	35–49	60	15

Residence	Debre Markos	288	71.8
	Machakel	37	9.2
	Gozamen	44	11
	Others	32	7.9

Occupation	Housewife	153	38.1
	Government employee	130	32.4
	NGO/private	80	20
	Others	38	9.5

Educational status	Illiterate	139	34.6
	Read/write	59	14.7
	Primary	46	11.5
	Secondary	62	15.5
	College/university	95	23.7

Husband education	Illiterate	101	25.2
	Literate	298	74.8

Income	<1000	72	18
	1000–2000	131	32.7
	2001–3000	88	21.9
	>3000	110	27.4

Knowledge of iodized salt	Good	278	69.3
	Poor	123	30.7

**Table 2:** Factors associated with visible goiter among pregnant women, Debre Markos Town, North West Ethiopia (*N* = 401).

Variable	Visible goiter		COR (95% CI)	AOR (95% CI)	*P* value
	Yes	No			
Age					
15–19	3 (20%)	12 (80%)	2.7 (0.57, 13)	1.9 (0.4, 10)	0.20
20–24	14 (11.7%)	105 (88.3%)	1.5 (0.5, 4.3)	1.3 (0.38, 3.6)	0.35
25–29	14 (9.7%)	130 (90.3%)	1.2 (0.41, 3.4)	1.0 (0.29, 2.5)	0.54
30–34	6 (9.5%)	57 (90.5%)	1.1 (0.33, 4.0)	0.9 (0.24, 2.7)	0.66
35–49	5 (8.3%)	55 (91.7%)	1.00	1.00	
Educational status					
Illiterate	23 (16.5%)	116 (83.5%)	2.5 (1.3, 4.8)^*∗*^	1.5 (0.7, 3.3)	0.28
Literate	19 (7.2%)	243 (92.8%)	1.00	1.00	
Husband education					
Illiterate	20 (19.8%)	81 (80.2%)	3.1 (1.6, 5.9)^*∗*^	1.0 (0.4, 2.5)	0.90
Literate	22 (7.4%)	276 (92.6%)	1.00	1.00	
Income					
<1000ETB	13 (18.1%)	59 (81.9%)	**7.8 (2.1, 28.6)** ^*∗*^	**4.5 (1.1, 18.7)** ^*∗*^	**0.03**
1000–2000	19 (14.5%)	112 (85.5%)	6.0 (1.7, 21)	4.0 (1.0, 14.8)	0.50
2001–3000	7 (8%)	81 (92%)	3.1 (0.77, 12)	2.1 (0.5, 10.3)	0.80
≥3000ETB	3 (2.7%)	107 (97.3%)	1.00	1.00	
Cabbage consumption					
Yes	40 (12.4%)	282 (87.6%)	**5.5 (1.3, 23.1)** ^*∗*^	**5.2 (1.2, 22.3)** ^*∗*^	**0.02**
No	2 (2.5%)	77 (97.5%)	1.00	1.00	
Family size					
1–4	21 (7.8%)	247 (92.2%)	1.00	1.00	
4^+^	21 (15.8%)	112 (84.2%)	2.2 (1.2, 4.2)^*∗*^	1.6 (0.7, 3.4)	0.24
Millet consumption					
Yes	31 (13%)	207 (87%)	2.1 (1.01, 4.2)^*∗*^	1.0 (0.4, 2.3)	0.9
No	11 (6.7%)	152 (93.3%)	1.00	1.00	
Knowledge of iodized salt					
Good knowledge	16 (5.8%)	262 (84.2%)	1.00	1.00	
Poor knowledge	26 (21.1%)	97 (78.9%)	**4.4 (2.3, 8.5)** ^*∗*^	**2.4 (1.1, 5.2)** ^*∗*^	**0.03**
Sweet potato consumption					
Yes	41 (11.7%)	309 (88.3%)	6.6 (0.9, 49.3)	3.2 (0. 2, 15)	0.08
No	1 (2.0%)	50 (98%)	1.00	1.00	

^*∗*^Statistically significant at *P* < 0.05.

## Data Availability

The [visible goiter in SPSS] data used to support the findings of this study are available from the corresponding author upon request.

## Additional Points

Nutrition education on micronutrients by government (through health extension workers) and nongovernmental organizations, especially on iodine, and increasing knowledge of the community on the benefits of consuming iodized salt through information communication and education (ICE) activities should be emphasized. Including low-income household women in safety net programs by government is recommended to enhance their economy, thereby having access to a variety of iodine-rich diversified diets. Communities should use iodized salt for preparing meal rather than using local/bare salt. Women in the reproductive age group need to be made aware of the importance of consumption of adequately iodized salt. It needs a further intensive study to reach the definite cause of visible goiter among pregnant women which should include determination of urinary iodine concentrations recommended by the World Health Organization as pregnant women are the most vulnerable groups confronted by this problem.
